# Safety of ultra-permissive anemia within a cardiac surgery patient blood management program

**DOI:** 10.1016/j.xjon.2025.07.001

**Published:** 2025-07-14

**Authors:** Rawn Salenger, Michael C. Grant, Clifford E. Fonner, Amanda Rea, Charlie Evans, Linda F. Barr, R.C. Stewart Finney, Daniel T. Engelman

**Affiliations:** a Department of Surgery, University of Maryland School of Medicine, Baltimore, Md; b Division of Cardiac Surgery, University of Maryland St Joseph Medical Center, Towson, Md; c Department of Anesthesiology and Critical Care Medicine, The Johns Hopkins University School of Medicine, Baltimore, Md; d Maryland Cardiac Surgery Quality Initiative, Baltimore, Md; e Division of Pulmonary and Critical Care Medicine, The Johns Hopkins University School of Medicine, Baltimore, Md; f Department of Surgery, Baystate Medical Center, University of Massachusetts Chan Medical School-Baystate, Springfield, Mass

**Keywords:** anemia, coronary bypass surgery, patient blood management

## Abstract

**Objectives:**

There remains a lack of studies assessing the safety of highly restrictive patient blood management in cardiac surgery. Our patient blood management program has been focused on ultra-permissive anemia, tolerating hemoglobin concentrations 6.0 g/dL or more in nonbleeding patients. We reviewed our results following an ultra-permissive anemia strategy regarding blood transfusion rates and the association with major complications after cardiac surgery.

**Methods:**

Consecutive patients undergoing coronary artery bypass grafting managed with ultra-permissive anemia were compared with historical controls, labeled the pre–ultra-permissive anemia group, who were transfused at the discretion of the clinician. A 1:1 propensity score matching was performed, and the groups were compared for blood transfusion rates, major complications, length of stay, and cost.

**Results:**

A total of 1216 patients were analyzed. Patients in the ultra-permissive anemia group received significantly less packed red blood cells and other blood components. The mean intraoperative and postoperative packed red blood cells transfusion rates were significantly lower in the ultra-permissive anemia cohort, 2% and 12% versus 27% and 29% pre–ultra-permissive anemia (*P < .*001). Postoperative length of stay was reduced for patients with ultra-permissive anemia (6.0 days vs 7.3, *P < .*001). Early extubation rates were higher for patients with ultra-permissive anemia (78% vs 53%, *P < .*001). The incidence of stage 1 acute kidney injury was lower for patients with ultra-permissive anemia compared with pre–ultra-permissive anemia (20% vs 26%, *P = .*049) as were rates of atrial fibrillation (34% vs 41%, *P = .*013). All other major complications, including mortality, were similar.

**Conclusions:**

Our data suggest that ultra-permissive anemia, tolerating hemoglobin values as low as 6 g/dL, is safe and significantly reduces overall blood use.

**Figure:**
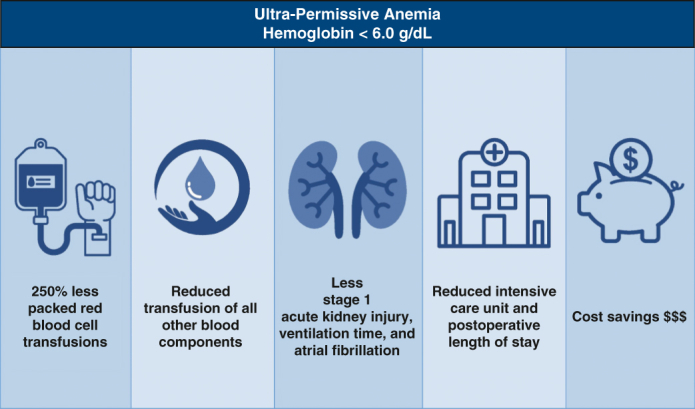
UPA, tolerating an Hgb level down to 6.0 g/dL, was found to be safe after cardiac surgery and associated with reduced transfusion, excellent outcomes, and cost savings.

**Central Message**

UPA, tolerating Hgb levels down to 6.0 g/dL, during and after cardiac surgery is safe, cost-effective, and associated with excellent clinical outcomes.

**Perspective**

The current Hgb threshold for permissive anemia after cardiac surgery is 7.5 g/dL, We studied an UPA strategy, tolerating Hgb 6.0 g/dL or greater. UPA was associated with a 250% reduction in red cell transfusions, more than $1 million saved over 3 years, and reduced mechanical ventilation time, LOS, atrial fibrillation, and stage 1 AKI.

The current benchmark for a restrictive transfusion strategy was set by the TRICS III Trial, which demonstrated that tolerating hemoglobin (Hgb) levels of 7.5 g/dL was safe after cardiac surgery.^1^ Numerous other studies also have failed to demonstrate inferior outcomes with restrictive blood transfusion protocols.^1^, ^2^, ^3^, ^4^, ^5^, ^6^, ^7^, ^8^ Transfusion of packed red blood cells (PRBCs) after cardiac surgery has been independently associated with major morbidity, including increased incidence of stroke, renal failure, infection, pulmonary complications, prolonged length of stay (LOS), and short- and long-term mortality.^3^^,^^4^^,^^7^, ^8^, ^9^, ^10^, ^11^, ^12^, ^13^, ^14^, ^15^ However, the elevated risk associated with perioperative anemia has been well documented, and the lowest safe Hgb level remains unknown.^3^^,^^4^^,^^16^, ^17^, ^18^ There remains a lack of published studies assessing the safety of highly restrictive patient blood management (PBM) protocols accepting more profound anemia. In 2015, our program implemented a PBM program heavily focused on ultra-permissive anemia (UPA) with a standard of no PRBC transfusions for nonbleeding patients with an Hgb concentration 6.0 g/dL or greater. We have reviewed our results following a UPA strategy regarding blood transfusion rates, major complications, LOS, and cost after cardiac surgery.

## Patients and Methods

### Patient Selection and Definitions

**Figure 1:**
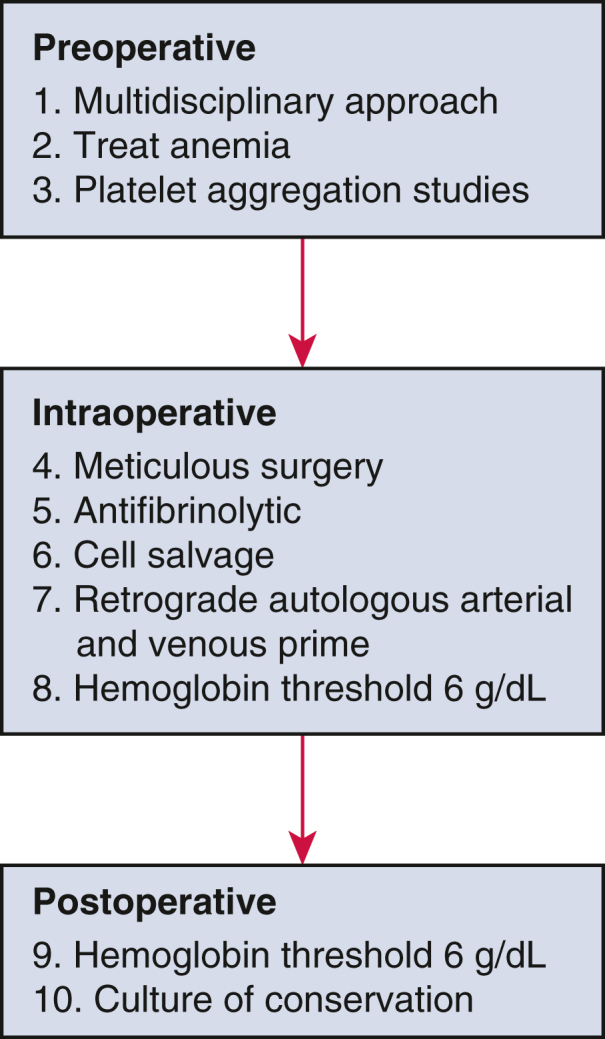
Ten-step PBM: displaying the 10 essential components of our program to reduce blood use.

Our analysis included consecutive adult patients undergoing isolated on-pump coronary artery bypass grafting (CABG). The UPA group comprised patients from January 1, 2019, to December 31, 2021, managed with our PBM program and UPA (transfusion considered only for Hgb < 6.0 g/dL in nonbleeding patients). Matched historical control patients, labeled the pre-UPA group, included patients from January 1, 2012, to December 31, 2014, and were transfused variably at the discretion of the bedside clinician. Since 2015, our PBM program has been heavily focused on UPA and restrictive transfusion thresholds (Figure 1). Details of our PBM program have been published.^19^^,^^20^ Briefly, we consider transfusion for patients with an Hgb less than 6.0 g/dL and a persistent metabolic acidosis or elevated serum lactate. If the inadequate oxygen delivery is also due to low cardiac index, we will attempt to correct the cardiac index to greater than 2.0 mL/min/m^2^. This will occur before, or concurrently, with deciding to transfuse, depending on the overall clinical picture. Actively bleeding patients, as defined by the perioperative team, are transfused with PRBC at the discretion of surgeon. Notably, there were no programmatic changes to anesthesia techniques or cardiopulmonary bypass management during the study time frame.

The primary outcomes analyzed to demonstrate the safety of UPA were operative mortality, stroke, and acute kidney injury (AKI). Secondary outcomes included early extubation, new postoperative atrial fibrillation, blood use, and LOS. All variables and outcomes except AKI were defined according to the Society of Thoracic Surgeons (STS) Adult Cardiac Surgery Database definition. AKI was defined according to Kidney Disease: Improving Global Outcomes criteria. The need for individual patient consent was waived by our Institutional Review Board (HP-00091144: approved 6/16/2020).

### Statistical Methods

To address potential biases in baseline characteristics between groups, a 1:1 propensity score matching was performed without replacement. Propensity scores were calculated using a logistic regression model including the following covariates: patient age, previous coronary artery bypass grafting surgery, congestive heart failure, dual antiplatelet therapy, anticoagulation, preoperative hematocrit, ejection fraction, urgent/emergency surgery status, STS Adult Cardiac Surgery Database Predicted Risk of Mortality, and preoperative intra-aortic balloon pump. Mean and frequency imputations were used in case of covariate missing data. This was used in only 2 patients because the continuous variables were 99.9% nonmissing and categorical variables were 100% complete. Patients within the control group were matched to patients from the treated/exposed group by nearest neighbor matching with a caliper value of 0.2 of the pooled SD of the logs of the propensity score. Standardized mean differences (SMDs) were calculated to compare baseline characteristics after matching (Table E1). A postmatching SMD below 0.1 was considered an acceptable difference. The common support assumption was assessed using the Kolmogorov–Smirnov nonparametric test. Common support intervals were determined using the trimming method and kernel density estimators. The threshold was set at 0.001. Statistical analysis was performed with EasyMedStat (version 3.38; www.easymedstat.com).

## Results

In total, 1216 patients were analyzed with 608 matched patients in each group. After matching, the UPA and pre-UPA groups had similar preoperative characteristics (Table 1). Age, left ventricular ejection fraction, preoperative hematocrit, and elective case status were similar for both cohorts. Mean STS predicted risk of mortality was 1.6 for the UPA group and 1.7 pre-UPA patients (*P = .*921). Operative details, including the number of bypass grafts, were also similar for both groups (Table 2). Unmatched data are provided in Table E2.

**Table 1:** Matched patient characteristics pre–ultra-permissive anemia versus ultra-permissive anemia

Variable	Pre-UPA n = 608 (%)	UPA n = 608 (%)	*P* value
Patient age	65	65	.937
Previous CABG	4 (<1)	6 (1)	.753
Congestive heart failure	150 (25)	136 (22)	.379
Dual antiplatelet therapy	113 (19)	96 (16)	.224
Anticoagulation therapy	268 (44)	297 (49)	.107
Preoperative hematocrit (mean)	39	39	.112
Ejection fraction	53	53	.718
STS Predicted Risk of Mortality (%) (mean, median, IQR)	1.7, 0.97, 0.52-2.0	1.6, 0.92, 0.53-1.6	.921
Preoperative intra-aortic balloon pump	111 (18)	109 (18)	.941
Urgent/emergency	458 (75)	448 (74)	.554

*UPA,* Ultra-permissive anemia; *CABG,* Coronary artery bypass grafting; *STS,* Society of Thoracic Surgeons; *IQR,* interquartile range.

**Table 2:** Operative and postoperative details

Variable	Pre-UPA N = 608 (%)	UPAN = 608 (%)	*P* value
Intraoperative PRBC	163 (27)	11 (2)	<.001
Intraoperative platelets	127 (21)	50 (8)	<.001
Intraoperative fresh-frozen plasma	40 (7)	5 (1)	<.001
Intraoperative cryoprecipitate	3 (<1)	8 (1)	.224
Postoperative PRBC	177 (29)	75 (12)	<.001
Postoperative platelets	197 (32)	80 (13)	<.001
Postoperative fresh-frozen plasma	74 (12)	15 (2)	<.001
Postoperative cryoprecipitate	64 (11)	16 (3)	<.001
Nadir intraoperative hematocrit (mean)	22	25	<.001
Cardiopulmonary bypass time (min)	98	95	.054
Crossclamp time (min)	81	79	.037
Mean No. of grafts	3	3	---

*UPA,* Ultra-permissive anemia; *PRBC,* Packed red blood cells.

**Figure 2:**
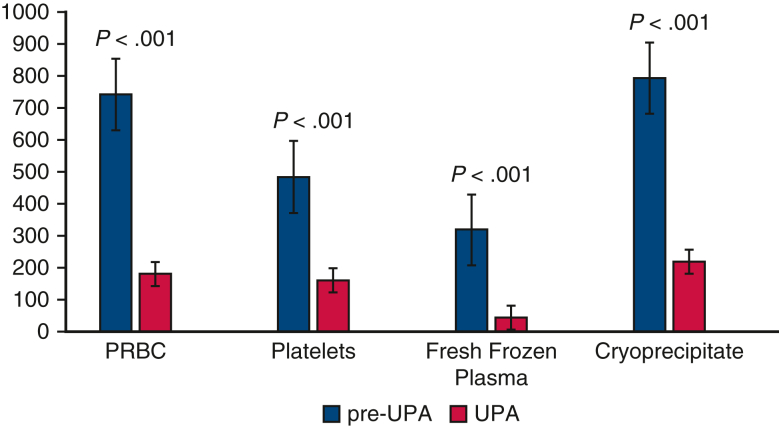
Blood use pre-UPA versus UPA groups demonstrates significantly lower transfusion rates for every blood component after implementation of UPA program. *PRBC*, Packed red blood cells; *UPA*, ultra-permissive anemia.

Patients treated according to PBM and UPA received less intraoperative and postoperative blood products, including PRBC, platelets, and fresh-frozen plasma (Table 2). The mean intraoperative and postoperative PRBC transfusion rates were significantly lower in the UPA cohort at 2% and 12% versus 27% and 29% for the pre-UPA group, respectively (*P < .*001). There were a total of 182 units PRBC transfused in the UPA group compared with 730 units PRBC administered to the pre-UPA patients. Transfusion rates of other blood components were also statistically lower in the UPA cohort throughout both phases of care (Figure 2). The UPA cohort had a significantly higher nadir hematocrit on cardiopulmonary bypass (25 vs 22, *P < .*001) compared with pre-UPA (Table 2). In the UPA group, the mean postoperative Hgb was 9.3 g/dL. Only 30 patients (5%) had postoperative Hgb less than 7.0 g/dL, and 15 patients had Hgb less than 6.5 g/dL in the UPA group. Postoperative Hgb values were not available in the STS Adult Cardiac Surgery Database during the pre-UPA period. Postoperative LOS was significantly shorter for the UPA cohort compared with pre-UPA (6.0 vs 7.3 days, *P < .*001), and intensive care LOS was modestly reduced for the UPA group, 51 versus 55 hours pre-UPA (*P < .*001). UPA was associated with higher rates of early extubation (78% vs 53%, *P < .*001) and reduced postoperative atrial fibrillation (34% vs 41%, *P = .*013). The incidence of stage 1 AKI was lower for UPA patients compared with pre-UPA (20% vs 26%, *P = .*049) with similar rates of stage 2 and 3 AKI. All other major complications, including operative mortality, were similar between groups (Table 3).

**Table 3:** Postoperative results

Variable	Pre-UPA N = 608 (%)	UPA N = 608 (%)	*P* value
Operative mortality	8 (1)	8 (1)	>.999
Stroke	10 (2)	6 (1)	.451
Reoperation for bleeding	9 (1)	12 (2)	.660
Stage 1 AKI	159 (26)	122 (20)	.014
Stage 2 AKI	14 (2)	7 (1)	.187
Stage 3 AKI	13 (2)	22 (4)	.170
Prolonged ventilation	47 (8)	36 (6)	.256
Early extubation	320 (53)	477 (78)	<.001
Postoperative atrial fibrillation	252 (41)	209 (34)	.013
Intensive care unit LOS	55 h	51 h	<.001
Postoperative LOS	7 d	6 d	<.001

*UPA,* Ultra-permissive anemia; *LOS,* length of stay.

### Cost Savings

PBM and UPA resulted in significant cost savings. Using the total transfusion numbers for the matched cohorts, an average number of units transfused per patient can be calculated. For PRBC, this was 1.2 units per patient in the pre-UPA group versus 0.3 units per patient in the UPA group, with a difference of 0.9 units per patient less in the UPA group. Applying this delta to the entire (unmatched) UPA cohort would yield a savings of $372,060 over 3 years. This calculation includes acquisition costs only (∼$200/unit PRBC), not the total cost for a transfusion.^25^, ^26^, ^27^ Added to this are the savings from the reduced use of other blood components for a total savings of $1,054,170 over 3 years (Table 4).

**Table 4:** Cost savings associated with ultra-permissive anemia (3 y)

Component	Pre-UPA units/patient (mean)	UPA units/patient (mean)	Delta	Acquisition cost/unit	Total savings for PBM group (n = 2067)
PRBC	1.2	0.3	0.9	$200	$372,060
Platelets	0.8	0.3	0.5	$500	$516,750
Fresh-frozen plasma	0.5	0.1	0.4	$50	$41,340
Cryoprecipitate	0.3	0.1	0.2	$300	$124,020
Total savings UPA group					$1,054,170

*UPA,* Ultra-permissive anemia; *PRBC,* Packed red blood cells.

## Discussion

### Safety of Ultra-Permissive Anemia

Our data demonstrate that managing patients with an UPA strategy as part of a PBM program is safe during and after isolated on-pump CABG surgery. UPA reduced red cell transfusions 250%, saved more than $1 million over 3 years, reduced ventilation time and LOS, and was associated with lower rates of AKI and atrial fibrillation. Only 5% of our total patients experienced a postoperative Hgb less than 7 g/dL; however, our programmatic focus on blood conservation helped reduce PRBC transfusions for patients overall, not only the most anemic patients. This reduction, along with decreased use of other blood components, was associated with similar and often improved outcomes. In addition to demonstrating the safety of UPA, our protocol was also associated with reduced rates of stage 1 AKI, time on mechanical ventilation, postoperative atrial fibrillation, intensive care unit stay, and postoperative LOS.

Although UPA may be a unique aspect of our PBM program, other elements of our program such as treatment of preoperative anemia, avoidance of hemodilution, and aggressive retrograde arterial and venous priming of our cardiopulmonary bypass circuit likely also played a role in reduced blood used (Figure 1). There was no formal change to general anesthesia or cardiopulmonary bypass management during this time; however, minimizing pre–cardiopulmonary bypass crystalloid administration and aggressive retrograde priming was emphasized as part of PBM. These elements of our PBM program may explain why nadir hematocrit on cardiopulmonary bypass was higher for UPA patients compared with controls (25 vs 22, *P < .*001). We have previously published data detailing our ultra-permissive strategy and the high compliance with the PBM protocol at our institution.^19^^,^^20^ According to our protocol, transfusion of PRBC was not considered in nonbleeding patients until Hgb values decreased to less than 6.0 g/dL. The safety of UPA has not, to our knowledge, been previously published.

### Comparison With Prior Trials

Our data do not provide evidence that UPA and lower transfusion rates resulted in improved outcomes in the UPA group. However, the practice of avoiding PRBC transfusion has been supported by a multitude of observational studies documenting the correlation between transfusion and increased major morbidity including renal failure, stroke, increased LOS, and mortality.^3^^,^^7^, ^8^, ^9^, ^10^, ^11^, ^12^, ^13^, ^14^ Similar to our data, a recent trial showed that anemia may be associated with reduced rates of postoperative atrial fibrillation, although mechanistic reasons for this finding are unknown.^4^ Additional support for permissive anemia comes from investigations demonstrating increased risk of postoperative events with the administration of even a single unit of PRBC after cardiac surgery.^9^^,^^11^^,^^21^ In fact, recent reviews of the STS *Adult Cardiac Surgery Database* showed that blood transfusion after cardiac surgery was also associated with increased risk of late mortality.^10^^,^^12^ Prior randomized trials that demonstrated the safety of a restrictive transfusion threshold have focused on higher Hgb concentrations than the current study (7.5-8.0 g/dL).^1^^,^^2^ These studies showed there is no benefit in transfusing patients above these values; however, they were not designed to examine the safety of more severe perioperative anemia. Our data add to the existing literature by suggesting that managing patients with the concept of UPA is safe after cardiac surgery down to an Hgb concentration of 6.0 g/dL.

### Anemia and Transfusion

A competing concern regarding restrictive transfusion practice is the risk associated with anemia during and after cardiac surgery. Anemic patients undergoing cardiac surgery have been shown to experience increased rates of renal failure, stroke, infectious complications, and mortality.^16^, ^17^, ^18^ The majority of these studies, however, are in patients who have preexisting preoperative anemia and likely represent a sicker group at baseline. Our PBM program attempts to minimize our number of anemic patients by treating preoperative anemia, avoiding hemodilution, and minimizing blood loss (Figure 1). When patients do become severely anemic, however, there is a lack of evidence to suggest that transfusing PRBC can mitigate the risks associated with anemia. On the contrary, our data indicate that a protocol of tolerating Hgb values as low as 6.0 g/dL reduces blood use without compromising outcomes. Prior studies have also raised concerns regarding severe anemia during cardiopulmonary bypass and the association with increased risk for AKI and neurologic complications.^22^^,^^23^ However, our data demonstrated no increase in neurologic or renal complications compared with matched historical controls (Table 3). In fact, patients managed with our UPA program experienced less stage 1 AKI. Blood transfusion is a known independent risk factor for AKI, and the lower transfusion rate may explain the decreased rates of AKI. More recent studies have suggested that oxygen delivery during cardiopulmonary bypass, and not simply Hgb concentration, is more closely linked to AKI risk.^20^^,^^24^ These same studies also show a correlation between blood transfusion and AKI.

Several investigators have sought to untangle the relative risks of anemia versus blood transfusion.^4^^,^^7^^,^^25^ A recent study used mediation analysis to quantitate the contributions of anemia or blood transfusion on outcomes after cardiac surgery. The study authors concluded that blood transfusions, not the anemia, accounted for the increased intensive care unit LOS, postoperative LOS, and higher mortality. Blood transfusion and anemia each individually contributed to increased risk for renal failure.^4^ A similar study examining more than 34,000 patients undergoing CABG from a statewide clinical database found that preoperative anemia was associated with a 4-fold increased risk of receiving a PRBC transfusion. After risk adjustment, this study also found that PRBC transfusion, not anemia, was significantly associated with postoperative renal failure, stroke, and mortality.^7^ The findings in these studies align with our results suggesting that UPA may be safer than transfusion in a nonbleeding anemic patient.

### Cost Impact

Reducing blood transfusions resulted in significant financial savings. Total cost savings from reduced acquisition costs alone over 3 years was more than $1 million. This calculation does not include the costs of processing or transfusing blood products, nor the resource savings associated with earlier extubation, lower rates of AKI, and reduced LOS experienced by the UPA group.^26^, ^27^, ^28^

### Limitations/Future Direction

Our trial was a retrospective cohort study with matched historical controls, subject to all the potential confounding variables and biases inherent in this type of study design. One significant weakness of our trial was the use of historical controls. Although we used propensity matching to ensure comparable groups, we cannot rule out the existence of unmatched confounding variables. Our propensity matching was focused on preoperative variables to demonstrate similar patients in each group. Although we did not match the groups on intraoperative variables, cardiopulmonary bypass time, crossclamp time, and mean number of bypass grafts were similar, implying there were no major intraoperative variability between groups. We also lacked a contemporaneous control group. This was partially because our entire program has adopted the concept of UPA, and this has become the standard at our institution. Therefore, we would have had difficulty convincing our clinicians to transfuse more liberally. The outcomes reported in this study with UPA could be due to several confounding variables, including general improvement in programmatic performance over time, surgical technique, or other perioperative enhancements, and therefore remain hypothesis generating. However, our trial was not designed to demonstrate improved outcomes, but the safety of UPA protocol with an Hgb threshold of 6.0 g/dL. A large randomized trial comparing UPA with a more conventional Hgb threshold remains an important area of future confirmatory research.

## Conclusions

Our data suggest that UPA, tolerating Hgb values as low as 6 g/dL, is safe and reduces overall blood use as part of a PBM program.

## Conflict of Interest Statement

R.S. is a consultant/advisor for Arthrex, Artivion, AtriCure, Encare, Zimmer Biomet, Grifols, and Innoviva, and receives research support from Innoviva. D.T.E. is on the Device Safety Monitoring Board for Edwards Lifesciences Transcatheter Valves, the Research Trial Steering Committees for Alexion, Genentech, Bayer, and RenalGuard, and is an advisor for Medela, Arthrex, AtriCure, Abiomed, Pharmacosmos. A.R. is a consultant for Edwards Lifesciences. All other authors reported no conflicts of interest.

The *Journal* policy requires editors and reviewers to disclose conflicts of interest and to decline handling or reviewing manuscripts for which they may have a conflict of interest. The editors and reviewers of this article have no conflicts of interest.

## Figures and Tables

**Table E1:** Standardized mean differences for each matched variable

Variable	Control N = 608	Treated/exposed N = 608	SMD	*P* value
Patient age, y	65.43 (±10.23)	65.46 (±9.37)	0.0025	.937
	Range (33.0-93.0)	Range (33.0-94.0)		
Previous CABG			−0.036	.753
Yes	4 (0.66%)	6 (0.99%)		
No	604 (99.34%)	602 (99.01%)		
Heart failure			0.054	.379
Yes	150 (24.67%)	136 (22.37%)		
No	458 (75.33%)	472 (77.63%)		
Dual antiplatelet therapy			0.074	.224
Yes	113 (18.59%)	96 (15.79%)		
No	495 (81.41%)	512 (84.21%)		
Anticoagulation			−0.096	.107
Yes	268 (44.08%)	297 (48.85%)		
No	340 (55.92%)	311 (51.15%)		
Hematocrit	38.86 (±5.3)	39.37 (±5.33)	0.096	.112
	Range (15.0-53.0)	Range (22.0-52.0)		
Ejection fraction	53.27 (±11.02)	53.44 (±10.44)	0.016	.718
	Range (13.0-79.0)	Range (13.0-71.0)		
STS PROM	0.0171 (±0.0242)	0.0155 (±0.0191)	−0.076	.921
	Range (0.00175-0.271)	Range (0.00124-0.228)		
IABP			0.0085	.941
Yes	111 (18.26%)	109 (17.93%)		
No	497 (81.74%)	499 (82.07%)		
Nonelective			0.038	.554
Yes	458 (75.33%)	448 (73.68%)		
No	150 (24.67%)	160 (26.32%)		

*CABG,* Coronary artery bypass grafting; *STS PROM,* Society of Thoracic Surgeons Predicted Risk of Mortality; *IABP,* intra-aortic balloon pump.

**Table E2:** Patient characteristics pre–ultra-permissive anemia versus ultra-permissive anemia unmatched

Variable	Pre-UPA n = 626 (%)	UPA n = 2067 (%)	*P* value
Patient age	66	67	.031
Previous CABG	9 (1.4)	7 (0.3)	.004
Congestive heart failure	161 (26)	335 (16)	<.001
Dual antiplatelet therapy	121 (19)	264 (13)	<.001
Anticoagulation therapy	274 (44)	1019 (49)	.017
Preoperative hematocrit	39	40	<.001
Urgent/emergency	473 (76)	1322 (64)	<.001
Preoperative intra-aortic balloon pump	113 (18)	287 (14)	.012
STS Predicted Risk of Mortality	1.8	1.4	.020
Ejection fraction	53	54	.954
Hypertension	545 (87)	1849 (89)	.11
Chronic obstructive pulmonary disease	80 (13)	396 (19)	<.001

*UPA,* Ultra-permissive anemia; *CABG,* Coronary artery bypass grafting; *STS,* Society of Thoracic Surgeons.
